# SimService: a lightweight library for building simulation services in Python

**DOI:** 10.1093/bioinformatics/btae009

**Published:** 2024-01-17

**Authors:** T J Sego

**Affiliations:** Department of Medicine, University of Florida, Gainesville, FL 32610-0225, United States

## Abstract

**Summary:**

Integrative biological modeling requires software infrastructure to launch, interconnect, and execute simulation software components without loss of functionality. SimService is a software library that enables deploying simulations in integrated applications as memory-isolated services with interactive proxy objects in the Python programming language. SimService supports customizing the interface of proxies so that simulation developers and users alike can tailor generated simulation instances according to model, method, and integrated application.

**Availability and implementation:**

SimService is written in Python, is freely available on GitHub under the MIT license at https://github.com/tjsego/simservice, and is available for download via the Python Package Index (package name “simservice”) and conda (package name “simservice” on the conda-forge channel).

Integrative biological models combine multiple submodels, which potentially use different modeling methods, operate at different scales, and/or are derived from different datasets, to incorporate the relevant complexity of a biological system or process (Agmon *et al.* 2022). Integrative biological modeling can leverage previous accomplishments from various domains by incorporating existing models, by combining them with new models that target additional data, and by using them in new ways as embedded or interacting systems. Combining biological models that are collaboratively developed in federated settings to target heterogeneous experimental and clinical data is likely a key technical capability for medical digital twin technologies (Laubenbacher *et al.* 2022), and recent work has developed a general framework to support such applications (Masison *et al.* 2021).

Executing multiple interconnected models should not require *de novo* development of one simulation that implements all models, which creates redundancy of effort by neglecting a wide variety of validated simulation software tools and is likely not reproducible (Karr *et al.* 2022). Rather, the execution of multiple models should result in *integrative simulation*, where multiple simulations are interconnected, executed and synchronized according to a schedule that reflects the dynamics of the models and how those dynamics are coupled. Effectively, integrative simulation should occur like software architectures in modern software design, where software libraries are integrated and interconnected through well-defined user interfaces to accomplish increasingly complex applications (e.g. a medical digital twin). For example, the integration of libRoadRunner (Somogyi *et al.* 2015), Antimony (Smith *et al.* 2009) and MaBoSS (Stoll *et al.* 2017) in CompuCell3D (Swat *et al.* 2012) supports applications such as explicit multicellular spatial modeling in physiologically based pharmacokinetic—pharmacodynamic modeling of drug uptake, distribution and metabolism (Sluka *et al.* 2016, Ferrari Gianlupi *et al.* 2022). Vivarium provides an interface to build and execute integrated simulations using user-defined implementations and supported simulators (Agmon *et al.* 2022). However, simulators are often designed as stand-alone applications with graphical and/or command-line user interfaces for the development and execution of stand-alone simulations, with little (if any) support for use as integrated software. For example, simulation frameworks like CompuCell3D and Tissue Forge (Sego *et al.* 2023) are stateful, preventing execution of multiple simulation instances in the same operating system (OS) process. In general, integrative simulation requires a well-defined way to launch, execute, interconnect, embed, and control the lifetime of, software components without loss of functionality or significant redesign of existing software.

This work proposes a solution to this need for integrative simulation, that simulations should be executed in separate, memory-isolated OS processes and controlled through proxy programming objects with a uniform, but extensible, execution interface. Similarly to solutions for isolated and reproducible software environments (e.g. by Docker and conda), this work proposes that each simulation should be executed in a container that allows the simulation both to execute as if it were a stand-alone application, and also to customize its container for providing a model- and simulation-specific interface with any application that might integrate it. This manuscript describes a software library, called *SimService*, that enables such a solution in the Python programming language. SimService provides an interface to generate interactive proxy objects of memory-isolated simulations, henceforth called *simulation services*. SimService is especially designed to encapsulate stateful simulations and frameworks for their integration in computational workflows and interconnection to accomplish integrative simulation. SimService supports customizing the interface of proxies at multiple levels of an underlying simulator, both statically in the SimService interface implementation of the simulator, as well as dynamically in the specification of a simulation.

## 1 Architecture Overview

**Figure 1.**
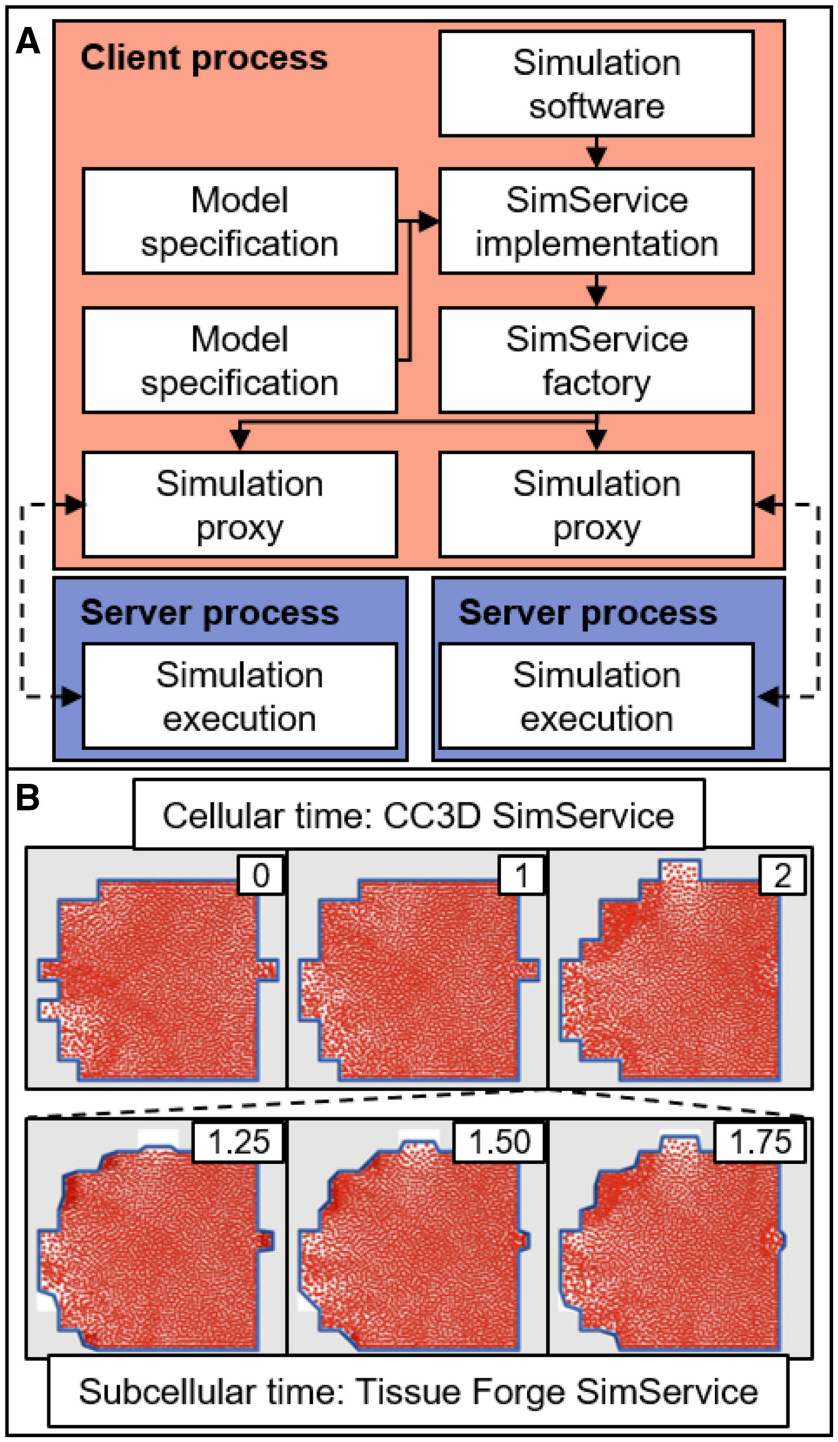
Overview of SimService applications. (A) Diagram of the pipeline for launching two concurrent simulations in the same process (i.e. the “Client process”) and corresponding processes that are launched for memory-isolated simulation execution (i.e. a “Server process”). Dashed lines indicate data throughput via MPI. (B) Application results coupling a CC3D simulation of cell motility with a Tissue Forge subcellular simulation of the cell membrane and cytoplasmic fluid using SimService implementations. CC3D integrates simulations in discrete time, whereas Tissue Forge integrates simulations in continuous time. In this case, multiple Tissue Forge substeps (bottom) are simulated per CC3D simulation step (top). Gray shaded locations indicate sites occupied by the medium and white locations indicate sites occupied by the cell according to CC3D. Particles inside the cell boundary represent parcels of fluid and particles constituting the cell boundary represent segments of the cell membrane according to Tissue Forge. Insets show time of results according to each simulator.

The overarching goal of SimService is to provide a noninvasive way to deploy a simulation as a Python object (i.e. a simulation service). SimService delivers this capability by providing an interface to define a simulation service. SimService uses information provided in an interface implementation to automatically create a process in which a simulation is launched, and a proxy object for interactive simulation execution, management and data throughput through a message passing interface (MPI, Fig. 1A). An interface implementation implements a single interface class that defines all operations to initialize, execute, interact with, and shut down a simulation. Registering an interface implementation with SimService provides the ability to launch the interface implementation as a simulation service, which is accomplished by instantiating the interface implementation in its own process (i.e. a server process) such that an interface implementation can perform all operations assuming an isolated memory space. The provider of a SimService implementation must ensure that system resources (e.g. graphics contexts) are appropriately acquired and released according to the functionality provided by their simulation service and the resources it uses.

When launching a simulation service, SimService constructs and returns a proxy object that facilitates interaction with the interface implementation instance. Most importantly, any methods or properties defined on an interface implementation in addition to the SimService interface are detected and added with the same signature to the interface of generated proxy objects such that a proxy object can be treated as if it were the interface implementation object. As such, implementations can arbitrarily add interface methods as relevant to underlying simulation features so long as all arguments and return objects can be serialized and deserialized.

A server process can make additional Python callables, whether functions or static, class or instance methods, available as methods on proxy objects with the same or a different name during runtime. For example, when a simulation framework executes custom routines in user-specified Python code, users can further customize the proxy interface of the simulation service instance according to their simulation or application. Such customizations, called *service functions*, are also implemented using the same MPI and can be made at any time during the lifetime of a simulation service.

End-users of a SimService implementation can create multiple instances of a simulation, destroy simulation instances at any time, and launch services provided by different implementations, from the same memory space. Proxy objects support serialization so that simulation services support parallel operations (e.g. batch execution). A simulation service can also generate, integrate, and store other simulation services, even to passing them through custom methods defined on its proxy object. The SimService interface provides basic methods that each simulation service must implement, which target procedures specific to a simulator or simulation. Methods that target an underlying simulation include performing startup and shutdown (e.g. allocating and releasing computing resources), and reporting available performance metrics. Methods that target a simulation include initializing the simulation (e.g. initializing data), starting (e.g. applying initial conditions) and stopping (e.g. storing final simulation data) the simulation, and integrating the simulation in time. Hence, while the interface of simulation services can vary significantly according to the underlying simulator and simulation, all simulation services provide the same methods for execution control.

As a simple example, consider a simulator of a 1D random walker. An implementation could define the simulator as in Listing 1.

**Listing 1**. A simple simulator of a 1D random walk in Python. Lines 4–13 define features for usage in other code. Lines 14–20 test the simulator when the script is executed.


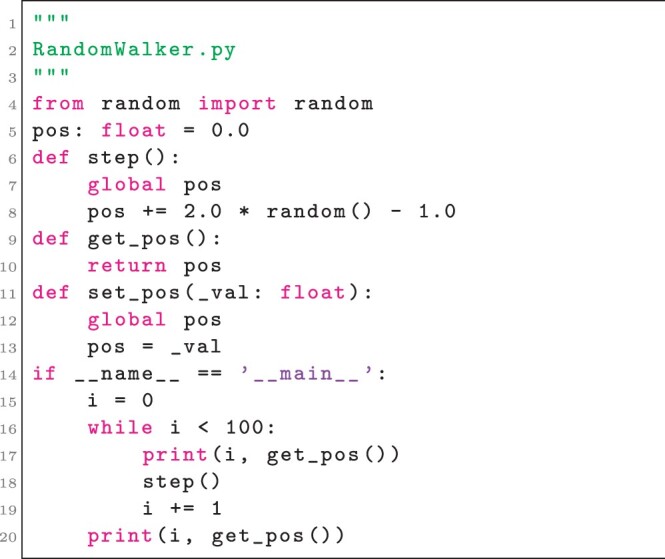


While the simulator in Listing 1 can be executed or imported and operated by other code, use of a module-level variable (i.e. the variable pos) prevents executing multiple instances of the simulator in the same process. A SimService interface implementation can be written on top of the simulator to generate memory-isolated simulator instances, as in Listing 2.

**Listing 2**. Implementation of the SimService interface for the simulator in Listing 1.


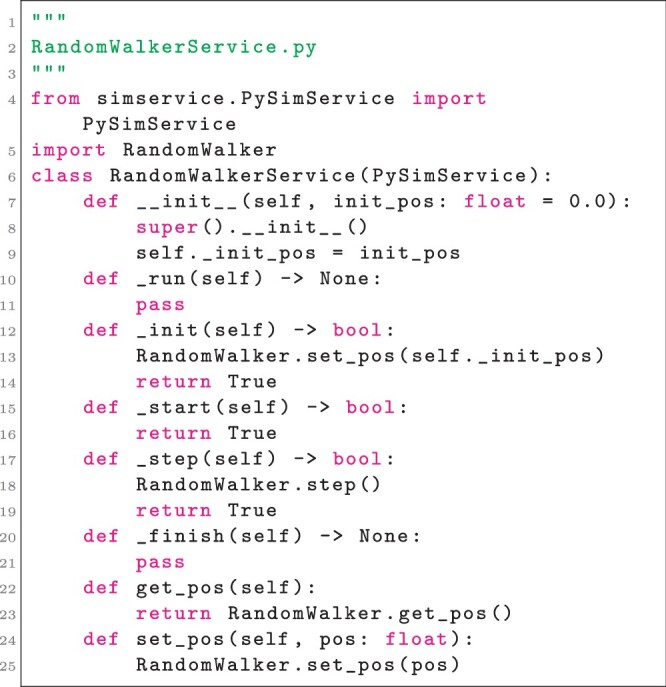


Note that in Listing 2, usage of the simulator is typical in the sense that no special care is taken to avoid collisions between multiple instances of the simulator. Note also that the methods _run, _init, _start, _step, and _finish are implementations of the SimService interface (i.e. PySimService), while the methods get_pos and set_pos are particular to the simulator. At the core of each simulation service is an instance of a PySimService implementation, which SimService will instantiate in its own process and provide a proxy that can be treated as if it were the PySimService implementation instance (i.e. a proxy to a RandomWalkerService instance has a method get_pos that is dispatched to, and returns the value of, the underlying get_pos method). Providing a registered SimService interface implementation and factory method for generating simulation services can be accomplished with mostly template code and only requires assigning a name to the simulation service, as in Listing 3.

**Listing 3**. Registering the SimService interface implementation defined in Listing 2 and providing a factory method to create simulation services. A new simulation service is created and returned each time the function at line 13 is called.


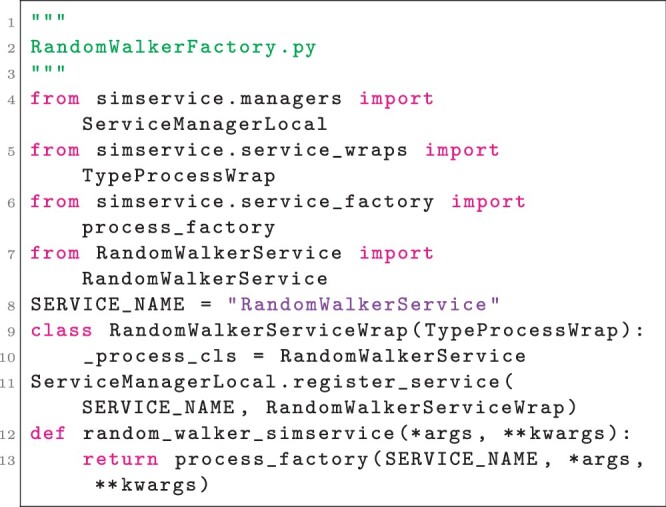


The factory method random_walker_simservice is a typical pattern for end-user applications in that it forwards constructor arguments for a PySimService implementation along with its associated name while providing a straightforward interface for the user. In the case of this simple example, no modifications to the simulator as defined in Listing 1 are required to allow creating and executing multiple simulator instances simultaneously, as in Listing 4.

**Listing 4**. Using the SimService interface implementation to create and orchestrate multiple simulators.


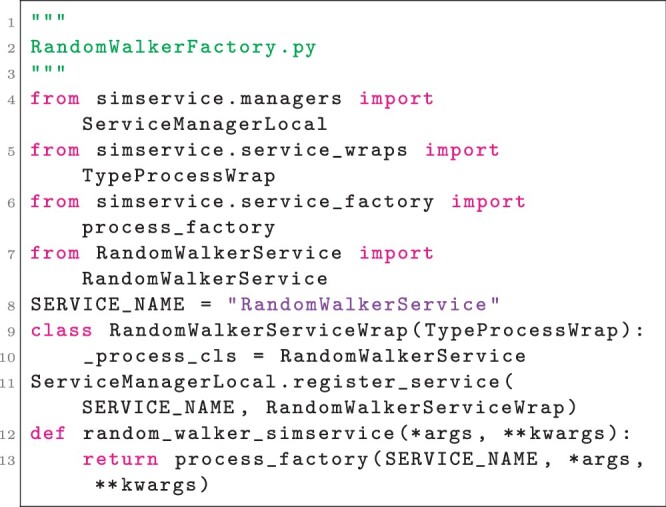


## 2 Applications

Simulation software that implements the SimService interface can provide user-facing methods that take model and/or simulation specification as input and return a SimService proxy to a memory-isolated simulation instance. Such proxies allow concurrent execution of multiple simulation instances (e.g. batch execution, multi-method simulation) and deployment of simulations in computational pipelines such as those that perform numerical sensitivity analysis, model calibration to given data, and control (An *et al.* 2017). This capability is well demonstrated using the SimService implementation distributed for CC3D (as of version 4.4.0). The CC3D SimService implementation provides a method to produce a CC3D simulation proxy with pre-defined data throughput, simulation monitoring features and support for CC3D users to add model-specific proxy interface features from within their simulation specification (i.e. CC3D user-defined service functions). The CC3D SimService implementation is also the backend that enables CC3D interactive execution in Jupyter Notebook (Chung *et al.* 2023). Simulation software that provides a Python API also allows *ad hoc* SimService implementations for generating integrated simulation software components.

For example, cells according to CC3D are volume-excluding objects on a regular lattice and typically represented as spatially homogeneous, whereas Tissue Forge provides features for lattice-free, particle-based modeling of both solids and fluids. A Tissue Forge simulation can construct particle-based representations of subcellular objects like the cell membrane and cytoplasmic fluid and simulate those subcellular dynamics as the cell membrane is interpolated between two given cellular domains. A Tissue Forge SimService implementation of such a simulation can then dynamically provide an interface to upload new cellular configurations and simulate subcellular dynamics along with a simulation that generates new cellular configurations, such as a CC3D SimService implementation. In this case, an orchestrating simulation would instantiate a multicellular simulation as a CC3D SimService and a partner subcellular simulation as a Tissue Forge SimService, integrate the CC3D SimService instance in time, forward an updated cellular configuration from the CC3D SimService to the Tissue Forge SimService instance, and then integrate the Tissue Forge SimService instance to update the subcellular simulation (Fig. 1B). Note that such an application introduces differences in the rate of model dynamics, where the subcellular simulation accomplishes multiple simulation steps per simulation step of the multicellular simulation. SimService allows an implementation to handle such differences in multiple ways, where a request to integrate a simulator through its proxy can correspond to a single step of the simulator, or to multiple steps as specified through custom interface methods (e.g. simulation time per step). For more details on the simulation shown in Fig. 1B and access to its source code, see https://github.com/tjsego/simservice/tree/main/examples/ExplicitCell.

CC3D provides additional applications of interest using SimService for integrative modeling and simulation. First, embedded models have become commonplace in multicellular simulation to describe subcellular and/or systems state dynamics, such as when modeling *in vivo* and *in vitro* viral infection, immune response and drug dosing (Sego *et al.* 2020, Aponte-Serrano *et al.* 2021, Ferrari Gianlupi *et al.* 2022, Sego *et al.* 2022) in CC3D using ordinary differential equation models specified in Antimony or the Systems Biology Markup Language and executed in libRoadRunner (Smith *et al.* 2009, Somogyi *et al.* 2015, Keating *et al.* 2020). While libRoadRunner supports launching multiple simulation instances from the same process, SimService enables providing the same capability for frameworks without such support such that, e.g. a CC3D simulation can embed a model of subcellular particle dynamics executed in other software like HOOMD-blue as a simulation service (Anderson *et al.* 2019). The same can be generally said for integrating software packages that provide command-line interfaces [e.g. Cyto-Sim (Sedwards *et al.* 2007), MEDYAN (Popov *et al.* 2016)] and Docker images, where a SimService interface implementation can automate the procedures of launching command-line tools or containers, passing input information, and retrieving and storing simulation output, in expressive SimService interface methods defined on simulation service proxies. In this way, SimService is a supplement to, rather than a replacement for, container-based solutions. Second, simulations of subdomains of a biological system that interact over long distances (e.g. a site of infection and lymph node) can be independently developed, concurrently executed as simulation services and orchestrated to interact (e.g. immune cell recruitment) through model-specific interface customization. Hence, SimService provides a straightforward way for modelers to share their simulations, and for other modelers to use those simulations, as integrated software components.

## 3 Availability and future directions

SimService enables rapid development of memory-isolated simulation services with the necessary flexibility to support applications by both software developers and end users in integrative biological simulation. Such capability is especially relevant to related work where interoperable simulations can now be assembled and executed as simulation services through implementations of the SimService interface. SimService allows necessary customization of a simulation service interface for particular simulations while providing the necessary generality to promote scalable integrative simulation frameworks. SimService could also act as an enabling technology to improve accessibility and reproducibility by providing a means to generalize simulation execution in projects like BioSimulators (Shaikh *et al.* 2022) and BioModels (Le Novère *et al.* 2006). In such cases, infrastructure could provide interfaces developed from the SimService interface that also enforce compliance with standards like SED-ML (Waltemath *et al.* 2011) such that execution and deployment of simulation components (e.g. those that implement the specifications for SED-ML Simulation, DataGenerator, and Output, per SED-ML Level 1 Version 4) in Python are both generalized. To this end, future work will provide additional support for heterogeneous computing environments and remote computing services to support large-scale simulations like those enabled by Vivarium (Agmon *et al.* 2022), and performance optimizations for locally executed simulation services (e.g. data-intensive message passing through shared memory). Future work using SimService should implement model and data standards supporting interoperability, reproducible simulations and workflows, and translation of data between scales and disparate modeling methodologies.

SimService source code is freely available on GitHub under the MIT license at https://github.com/tjsego/simservice, and is distributed via the Python Package Index (package name ”simservice”) and conda (package name ”simservice” on the conda-forge channel). Documentation supporting development of a SimService interface implementation and basic usage, as well as documentation of the entire SimService Python module, is publicly available at https://simservice.readthedocs.io.

## Data Availability

The source code for SimService is publicly available at https://github.com/tjsego/simservice.
